# Re-orienting in space: do animals use global or local geometry strategies?

**DOI:** 10.1098/rsbl.2010.1024

**Published:** 2010-12-15

**Authors:** Debbie M. Kelly, Cinzia Chiandetti, Giorgio Vallortigara

**Affiliations:** 1 Department of Psychology, University of Manitoba, 401 Duff Roblin, Winnipeg, Manitoba, CanadaR3T 2N2; 2 Centre for Mind/Brain Sciences, University of Trento, Rovereto, Italy; 3 Department of Psychology, University of Trieste, Trieste, Italy

**Keywords:** orientation, geometric encoding, domestic chick, racing pigeon

## Abstract

Here we compare whether birds encode surface geometry using principal axes, medial axes or local geometry. Birds were trained to locate hidden food in two geometrically identical corners of a rectangular arena and subsequently tested in an L-shaped arena. The chicks showed a primary local geometry strategy, and a secondary medial axes strategy, whereas the pigeons showed a medial axes strategy. Neither species showed behaviour supportive of the use of principal axes. This is, to our knowledge, the first study to directly examine these three current theories of geometric encoding.

## 1. Introduction

The first step for navigation is determining which direction to begin travelling—to orient. Visual-based environmental cues can be categorized into features (e.g. colour or pattern) or geometry (e.g. distance or direction). Studies suggest that these cues are processed differently; features being learned associatively, whereas geometry is encoded incidentally [1]. Given that geometric cues are encoded by all species studied thus far and that encoding occurs even when distinctive, and often more informative, features are available, it is important to understand how geometry is extracted from an environment. Geometric cues must be encoded in a way that allows for reliable orientation but is not computationally demanding [2]. Initially it was thought that animals encode the principal axes of an environment [3]. Although principal axes would allow for an economical means of computing heading, they do not retain information about the environmental shape and are thus not informative enough for use in complex environments. Other strategies have been suggested but they have either been shown unnecessary (more parsimonious alternatives could be used) or have not been examined empirically [4].

**Figure 1.**
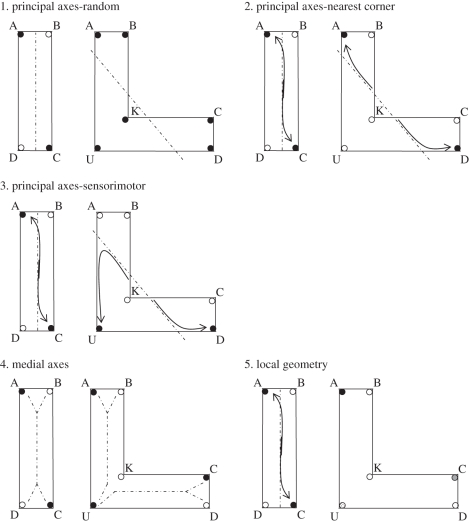
Schematic of rectangular training environment and L-shaped testing environment. Birds were trained to locate food hidden at geometrically equivalent corners (corners A and C are used as examples). The birds were subsequently tested in an L-shaped environment that allowed for the testing of each of the three main hypotheses. The filled circles indicate the corners where the birds should search during testing, whereas open circles represent corners that are predicted not be chosen.

The main goal of our study was to examine how animals encode surface geometry. Two avian species, chicks and pigeons, were chosen, as a great deal is known about their spatial abilities. The birds were trained to locate food hidden at two corners within a rectangular arena. During testing, the environment was modified to differentiate among possible encoding strategies. This L-shaped environment allowed us to examine whether the birds were responding according to principal axes, medial axes or a local geometry strategy—the principal axis is shifted in this new environment to continue through the centroid of the shape [4]. Figure 1 shows schematics of the training and testing environments.

If the birds were using principal axes there are three hypotheses as to how this strategy might be observed: (i) a random hypothesis would state that because neither the primary nor secondary axes would lead directly to a goal location, the birds should choose randomly among the corners, (ii) a nearest corner hypothesis would propose that the birds follow the principal axes until they meet with a wall and then choose the closest corner [4], or (iii) the birds might follow the principal axes until they meet with a wall and then adopt a sensorimotor routine of turning in the trained direction along the wall.

If the birds were using medial axes, hypothetical trunks would be centred down the length of the arena's arms, and branch out to each of the corners. We would expect that birds would follow the trunks down the arms and then diverge along the branch with the correct sense information (left or right) as learned in training.

Finally, if the birds were using a local geometry strategy, each corner presents a unique combination of geometric information that could be coupled with sense information learned during training. One corner of the environment is correct according to both sense and absolute geometrics; also, one corner is correct according to both sense and relative metrics. Given that both species encode absolute and relative metrics [5,6], we predict that the birds should choose these corners equally frequently. Importantly, the birds should show significantly less response to the corner that is subtended by one wall that is correct according to sense plus metrics and the other wall incorrect according to metrics (corner U, figure 1). Finally, we predict that the birds should show minimal response to the corner that has incorrect angular information and is subtended by two walls of equal length (corner K, figure 1).

## 2. Methods

### (a) Subjects

Subjects were 20 day-old male domestic chicks (*Gallus gallus*) and 12 (six male and six female) adult racing pigeons (*Columba livia*). The chicks were reared individually in cages (22 × 30 × 40 cm, width, height, depth) at a controlled temperature (30°C). Food and water were provided ad libitum. The pigeons were housed individually (46 × 76 × 61 cm, width, height, depth) at a controlled temperature (22°C), and a 12 L : 12 D cycle. The birds were maintained at 85 per cent of their ad libitum weight, with water and grit ad libitum.

### (b) Apparatus

The training apparatuses were uniformly coloured rectangular arenas (chicks: 80 × 20 × 45 cm, pigeons: 200 × 50 × 70 cm, length, width, height, respectively). Four identical containers were located, one in each corner (chicks: 4 × 4 cm, pigeons: 6.5 × 4 cm, diameter × height). The arenas were fully enclosed, with centrally mounted cameras.

The testing apparatuses were L-shaped arenas (the two longest and shortest walls were of the same length and height as in training; the intermediate walls were 60 cm long for chicks and 150 cm for pigeons). Six food containers were present, one in every corner—including the re-entrant corner (corner K, figure 1).

### (c) Experimental procedures

Both species were trained to locate food hidden inside two ‘correct’ containers (corners AC for group AC and corners BD for group BD).

#### (i) Chicks

One day before training, the chicks were deprived of food for 3 h and underwent a familiarization phase. Training began on the next day and continued for three consecutive days. Individual chicks were given three daily sessions of 10 trials each. Chicks were disoriented before each trial by being placed inside a box and rotated for 1 min at 10–12*g*. The disoriented chick was placed in the arena; a peck to one of the correct containers resulted in a food reward. After trial 30 if an incorrect container was chosen, the chick was removed from the arena and the next trial started. During the test phase, disoriented chicks were given one session with 10 non-reinforced test trials in the L-shaped arena. Only first choices were measured.

#### (ii) Pigeons

Pigeons underwent a familiarization phase before training. Disorientation procedures were the same as for the chicks. Training began on the next day and occurred in stages such that the birds learned to peck through paper towel secured over each of the tins. Only the correct container was rewarded. Each daily session consisted of 10 trials for five sessions. The final training phase consisted of five reinforced and five non-reinforced trials. The birds advanced to testing once they completed two consecutive sessions each with 80 per cent or greater first responses to the correct containers.

During the test phase, disoriented pigeons were given five sessions (one per day), consisting of five baseline trials in the rectangular arena and five test trials in the L-shaped arena. All test trials were non-reinforced. Only first choices were measured.

## 3. Results

There were no differences in the average errors made during training between the two groups (chicks: group AC: mean ± s.e.m.: 20 ± 1.27 and group BD: 21.6 ± 1.77; *t*_18_ = 0.735, *p* = 0.472; pigeons: group AC: mean ± s.e.m.: 5.17 ± 0.41 and group BD: 3.33 ± 0.27; *t*_10_ = 1.17, *p* = 0.269). By the completion of training for the chicks, and throughout testing for the pigeons, baseline performance never fell below 74 and 80 per cent, respectively.

**Figure 2.**
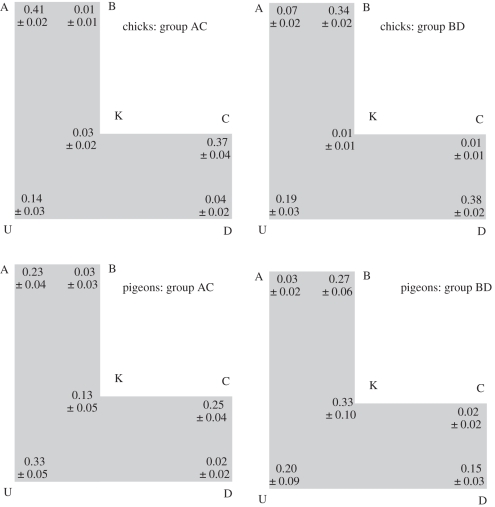
Means and s.e.m. for testing conditions.

### (a) Principal axes hypotheses

We examined whether the total number of choices among the six corners (shown in figure 2) differed from an equal distribution (16.67 choices per corner). A *χ*^2^ showed that the birds' choices of each corner were not evenly distributed (chicks: group AC: *χ*^2^_5_ = 111.411, *p* = 0 and group BD: *χ*^2^_5_ = 80.72, *p* = 0 and pigeons: group AC: *χ*^2^_5_ = 29, *p* = 0.00002 and group BD: *χ*^2^_5_ = 28.6, *p* = 0.00003). Thus, our results do not support the use of a random choice strategy.

To examine whether the birds were using a nearest corner strategy, we compared the total proportion of choices by both groups to corners A and D to chance (0.33). For the chicks, groups AC and BD chose corners A and D significantly more than expected by chance (group AC: *M* = 0.45, s.e. = 0.024; *t*_8_ = 5.367, *p* = 0.0005 and group BD: *M* = 45, s.e. = 0.037; *t*_8_ = 3.220, *p* = 0.010), but the division of choices was not equal between the two corners (group AC: *t*_8_ = 8.251, *p* < 0.0001 and group BD: *t*_8_ = 17.270, *p* < 0.0001); each group chose their training corner significantly more than the geometrically incorrect corner. For pigeons, group AC did not choose corners A and D significantly more than that expected by chance, whereas group BD chose these corners significantly *less* than that expected by chance (group AC: *M* = 0.25, s.e. = 0.04; *t*_4_ = −1.87, *p* = 0.12 and group BD: *M* = 0.18, s.e. = 0.04; *t*_4_ = −3.65, *p* = 0.015).

Finally, we examined whether the birds used principal axes along with a sensorimotor routine. Here we expected group differences with group AC choosing corners U and D, whereas group BD choosing corners U and A. Both groups of chicks chose the predicted corners significantly *less* than expected by chance (chance = 0.33; group AC: *M* = 0.18, s.e. = 0.036; *t*_8_ = −4.178, *p* = 0.002 and group BD: *M* = 0.26, s.e. = 0.016; *t*_8_ = −4.287, *p* = 0.002), whereas neither group of pigeons chose the predicted corners more than that expected by chance (group AC: *M* = 0.35, s.e. = 0.06; *t*_4_ = 0.36, *p* = 0.737 and group BD: *M* = 0.23, s.e. = 0.08; *t*_4_ = −1.20, *p* = 0.28).

### (b) Medial axes hypotheses

We compared the total proportion of choices of corners A, C and U by group AC to chance and the choices of corners B, D and U by group BD to chance. For chicks, groups AC and BD chose the expected corners more than chance (chance = 0.50; group AC: *M* = 0.92, s.e. = 0.025; *t*_8_ = 16.838, *p* < 0.0001 and group BD: *M* = 0.91, s.e. = 0.023; *t*_8_ = 17.571, *p* < 0.0001). A *χ*^2^ analysis showed that the chicks were not dividing their choices equally among the three corners (group AC: *χ*^2^_2_ = 13.848, *p* = 0.001 and group BD: *χ*^2^_2_ = 6.615, *p* = 0.037). This suggests that the chicks were not using the medial axes as a primary strategy.

For pigeons, group AC chose the expected corners more than that expected by chance, but group BD did not (chance = 0.50; group AC: *M* = 0.82, s.e. = 0.07; *t*_4_ = 4.84, *p* = 0.005 and group BD: *M* = 0.62, s.e. = 0.12; *t*_4_ = 0.93, *p* = 0.39). However, a *χ*^2^ analysis showed that the pigeons were dividing their choices equally among the three corners (group AC: *χ*^2^_2_ = 2.074, *p* = 0.355 and group BD: *χ*^2^_2_ = 3.516, *p* = 0.172; the pigeons make several errors to corner K—these choices cannot be accounted for by any of the hypotheses). This supports the hypothesis that both groups were using a medial axes strategy to reorient. Furthermore, we compared the proportion of choices made to the corner which contained all the correct local geometric information (corner A for group AC and corner D for group BD) to corner U which is subtended by one wall with the correct metric and sense (a long wall to the right) and one wall with incorrect metric and sense. Both groups chose these corners equally (group AC: *t*_4_ = −1.464, *p* = 0.203 and group BD: *t*_4_ = −0.791, *p* = 0.465); these results do not support a local geometric encoding strategy. Thus, both groups of pigeons showed clear reliance on medial axes to guide re-orientation.

### (c) Local cues hypotheses

To examine whether the chicks were using a local geometric strategy we compared the proportion of choices made to the corner which contained all the correct local geometry (corner A for group AC and corner D for group BD) to corner U which is subtended by one wall with correct metric and sense properties, and one wall with incorrect metric and sense properties. Both groups chose the corner that was correct according to local geometry significantly more than corner U or corner K (corner A versus corner U for group AC: *t*_8_ = 6.021, *p* = 0.0002 and corner A versus corner K for group AC: *t*_8_ = 6.021, *p* = 0.0002. Corner D versus corner U for group BD: *t*_8_ = 10.585, *p* < 0.00001 and Corner D versus corner K for group BD: *t*_8_ = 14.212, *p* < 0.0001). Further analysis showed that the birds used absolute and relative local geometry equally as neither group showed a significant difference between the two corners that were correct according to metrics (absolute or relative) and sense (corners A versus C for group AC: *t*_8_ = 0.840, *p* = 0.423 and corners B versus D for group BD: *t*_8_ =−1.078, *p* = 0.309). However, both groups of chicks showed systematic bias towards corner U, choosing it more than each of the other three incorrect corners (corner U versus B, K and D for group AC and corners U versus A, K and C for group BD, all *t*s > 2.57, *p*s < 0.05).

Thus, our data suggest that the chicks showed primary reliance on local geometry and a secondary reliance on medial axes. The pigeons, however, showed a strong reliance on medial axes by equally distributing their choices among the three correct corners according to a medial axes strategy.

## 4. Discussion

Both species learned to search at the two geometrically correct corners in a rectangular arena that lacked distinctive featural information. When tested in an L-shaped environment, neither species showed search behaviour supporting the use of principal axes. Chicks showed reliance on local geometrical cues primarily, with a secondary reliance on medial axes, whereas pigeons showed reliance on medial axes. Our empirical results indicate, to our knowledge, for the first time that principal axes are probably not used by reorienting animals, but rather medial axes or local geometry strategies best support our data. Species, age or ecological differences (e.g. flight may encourage encoding of global axes for pigeons and lack of flight, local cues for chicks), may guide how surface geometry is encoded, and these are all empirical issues deserving further examination. As all species studied to date show an encoding of geometric information, our method for directly comparing the three theories of geometric encoding will allow for a better understanding of how geometry is extracted from an environment.
